# Mitochondrial ROS regulate oxidative damage and mitophagy but not age-related muscle fiber atrophy

**DOI:** 10.1038/srep33944

**Published:** 2016-09-29

**Authors:** Giorgos K. Sakellariou, Timothy Pearson, Adam P. Lightfoot, Gareth A. Nye, Nicola Wells, Ifigeneia I. Giakoumaki, Aphrodite Vasilaki, Richard D. Griffiths, Malcolm J. Jackson, Anne McArdle

**Affiliations:** 1 MRC-Arthritis Research UK Centre for Integrated Research into Musculoskeletal Ageing, Department of Musculoskeletal Biology, Institute of Ageing and Chronic Disease, University of Liverpool, Liverpool, L7 8TX, U.K

## Abstract

Age-related loss of skeletal muscle mass and function is a major contributor to morbidity and has a profound effect on the quality of life of older people. The potential role of age-dependent mitochondrial dysfunction and cumulative oxidative stress as the underlying cause of muscle aging remains a controversial topic. Here we show that the pharmacological attenuation of age-related mitochondrial redox changes in muscle with SS31 is associated with some improvements in oxidative damage and mitophagy in muscles of old mice. However, this treatment failed to rescue the age-related muscle fiber atrophy associated with muscle atrophy and weakness. Collectively, these data imply that the muscle mitochondrial redox environment is not a key regulator of muscle fiber atrophy during sarcopenia but may play a key role in the decline of mitochondrial organelle integrity that occurs with muscle aging.

Skeletal muscle is a major site of metabolic activity and is the most abundant tissue in the human body^1^. Age-related muscle atrophy (sarcopenia) and weakness, characterized both by loss of lean muscle mass and reduced skeletal muscle function, is a major contributor to frailty and loss of independence in older people^2^. Studies of humans indicate that by the age of 70, there is a ~25–30% reduction in the cross sectional area (CSA) of skeletal muscle and a decline in muscle strength by ~30–40%^3^. Age-dependent loss of muscle mass and function has a complex aetiology and the primary biochemical and molecular mechanisms underlying this process have not been fully determined.

Oxidative stress has been suggested to be a key factor contributing to the initiation and progression of the muscle atrophy that occurs during aging^4^,^5^. Consistent with a role of oxidative stress as a contributor to sarcopenia, studies from our group^6^,^7^,^8^,^9^,^10^,^11^ and others^12^,^13^ have shown that genetic manipulations of redox regulatory systems can alter the aging process in muscle. Skeletal muscle decline with advancing age has been linked to an altered oxidative status of redox-responsive proteins^14^ and a number of studies have indicated a positive correlation between tissue concentration of oxidized macromolecules and life span including an increase in DNA damage^15^, accumulation of oxidized proteins^16^ and increased levels of lipid peroxidation^17^ with age. In support of these findings recent quantitative proteomic approaches^18^ have further provided evidence that muscle aging is associated with a reduction in redox-sensitive proteins involved in the generation of precursor metabolites and energy metabolism, implying age-related redox changes as an underlying cause of age-related muscle atrophy.

Skeletal muscle produces reactive oxygen and nitrogen species (RONS) from a variety of subcellular sites^5^,^19^ and there is evidence that isolated skeletal muscle mitochondria exhibit an age-related increase in hydrogen peroxide (H_2_O_2_) production^20^,^21^. Furthermore, muscle aging is associated with reduced mitochondrial oxidative-phosphorylation^22^,^23^, reduced mitochondrial DNA (mtDNA) content^24^,^25^, accumulation of mutated mtDNA^26^, impaired mitophagy^27^ and increased mitochondrial permeability transition pore sensitivity^28^, which are all proposed to contribute to the sarcopenic phenotype. Although cumulative oxidative stress has been proposed to induce age-associated reductions in mitochondrial function^29^,^30^, this remains a controversial topic^31^,^32^.

We^33^,^34^ and others^35^,^36^ have recently reported that pharmacological application of the mitochondria-targeted SS31 tetrapeptide can attenuate mitochondrial superoxide production in intact mitochondria of skeletal muscle fibers. This pharmacological approach complements genetic approaches, including those using targeted overexpression of the human catalase gene to mitochondria (MCat mice)^2^,^23^. Such pharamacological agents may have substantial translational implications for the use and/or development of mitochondria-targeted antioxidants for treatment of human mitochondrial myopathies as well as mtROS mediated muscular dysfunctions.

The purpose of the present study was to determine the effect of the mitochondria-targeted SS31 peptide on redox homeostasis in muscles of old mice, including mitochondrial ROS (mtROS) and oxidative damage, mitochondrial content and mitophagy and on age-related muscle atrophy and weakness. Through this approach we aimed to determine the role of modified mitochondrial redox homeostasis on age-related loss of muscle mass and function.

Our findings demonstrated that a reduction in mtROS in response to SS31 treatment prevented age-related mitochondrial oxidative damage and improved mitophagic potential, but further demonstrated that changes in mitochondrial redox environment towards a more reduced state failed to rescue the sarcopenic phenotype associated with muscle fiber atrophy and loss of muscle mass and strength. This work has therefore identified that the age-related changes in mitochondrial redox potential play a key role in the loss of mitochondrial organelle integrity that occurs with aging, but are not involved in the processes of age-related muscle fiber atrophy.

## Results

### Characterization of the age-related changes in muscle fibers indicate that atrophy is attributed to a reduction in fiber size of type IIb, IIx and IIa muscle fibers

**Figure 1:**
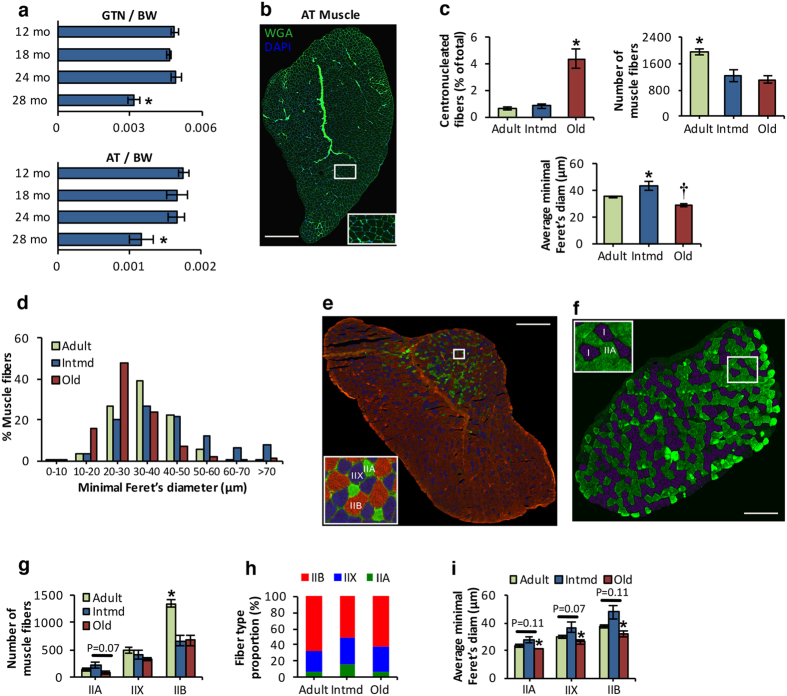
Sarcopenia is associated with type IIb, IIa and IIx myofiber atrophy. (**a**) Age-related changes in gastrocnemius (GTN) (top panel) and anterior tibialis (AT) muscle mass (bottom panel), normalized to body weight (BW). *P < 0.05 compared with values from the other age-groups, n = 6–8 mice/group. (**b**) Transverse section of an AT muscle from an adult mouse stained with wheat germ agglutinin (WGA, 5 μg/ml, green), to visualize extracellular matrix, and 4′,6-diamidino-2-phenylindole (DAPI, 1 μg/ml, blue), to mark nuclei and show fiber sizes and presence of centrally located nuclei. Scale bar, 600 μm. (**c**) Percentage of fibers showing centrally located nuclei in AT muscle from adult, intermediate (Intmd) and old mice (top left panel); total number of muscle fibers in AT muscle from adult, intmd and old mice (top right panel); mean minimal Feret’s diameter of individual fibers from AT muscle of adult, intmd and old mice (bottom panel). *P < 0.05 compared with values from the other age-groups, †P < 0.05 compared with values from adult mice, n = 4–6 mice/group. (**d**) Frequency distribution of fiber size of AT muscle from adult, intmd and old mice. n = 4–6 mice/group. (**e**) Representative triple immunofluorescent staining of myosin heavy chain (MHC) isoforms; MHC IIa (green), MHC IIx (blue) and MHC IIb (red) performed on an AT cryosection obtained from an adult mouse. Scale bar, 500 μm. (**f**) Representative immunofluorescent image of muscle fiber types; MHC I (purple) and MHC IIa (green) performed on a soleus cryosection obtained from an adult mouse. Scale bar, 150 μm. (**g**) Total number of MHC IIa, MHC IIx and MHC IIb muscle fibers in AT muscle of adult, intmd and old mice. *P < 0.05 compared with values from the other age-groups for the respective fiber type, n = 4–6 mice/group. (**h**) Histogram showing the fiber-type distribution of AT muscle from adult, intmd and old mice. n = 4–6 mice/group. (**i**) Quantification of MHC IIa, MHC IIx and MHC IIb muscle fiber size of AT muscle from adult, intmd and old mice. *P < 0.05 compared with values from intmd mice, n = 4–6 mice/group. Statistical differences between groups were determined using analysis of variance (ANOVA) followed by LSD *post hoc* test. Data represent mean ± s.e.m.

To determine the time-course of age-related phenotypic changes that occur in skeletal muscle, we initially assessed gastrocnemius (GTN) and anterior tibialis (AT) muscle mass in 12, 18, 24 and 28 month old mice. Both muscles retained mass until 24 months of age, but showed age-related sarcopenic changes (~33% reduction in mass relative to body weight) at 28 mo of age. (Fig. 1a). Histological analysis of AT muscle (Fig. 1b) from adult (10–11 mo) intermediate (intmd, 18–19 mo) and old (28 mo) mice revealed a substantial number of centrally nucleated fibers in AT muscle of old mice (Fig. 1c), which reflects previous cycles of degeneration and regeneration^8^. Total number of fibers per AT muscle was reduced by a mean of 37% and 43% in intmd and old mice respectively in comparison with adult mice (Fig. 1c), indicating a significant loss of muscle fibers between adult and intermediate ages, which had no effect on muscle mass (Fig. 1a). We then determined whether the AT muscle of intermediate mice showed compensatory hypertrophy of the remaining muscle fibers (Fig. 1c). The average minimal Feret’s diameter of muscle fibers increased significantly from adult to intermediate ages, followed by a significant reduction at 28 mo old (Fig. 1c) demonstrating an age-related hypertrophy followed by an atrophy. To further determine whether the age-related decline in muscle mass (Fig. 1a) coincided with myofiber atrophy, we undertook quantitative analysis of individual fiber size and revealed that myofiber size distribution shifted to the left in the old mice, suggesting that smaller fibers are more prevalent in muscle of 28 month old mice (Fig. 1d). Moreover, significant differences in myofiber distribution were also observed between adult and intmd mice; ~27% of total fibers per AT muscle from intmd mice showed a minimal Feret’s diameter of >50 μm in comparison with ~7% of fibers of adult mice (Fig. 1d). In order to define whether a fiber type specificity in muscle atrophy existed, AT cross sections were immunolabeled for the 4 myosin heavy chain (MHC) isoforms (Fig. 1e). AT muscle expressed MHC IIa, IIx and IIb fibers (Fig. 1e), with no evidence for MHC I expression, in contrast to the soleus muscle (Fig. 1f). A selective loss of type IIb fibers was seen in the AT muscle between adult and intermediate ages (Fig. 1g,h) with no further reduction with increasing age. Fiber type size analyses revealed that the sarcopenic phenotype observed in old mice was associated with a significant reduction in IIa, IIx and IIb myofiber size (Fig. 1i) indicating that age-related muscle atrophy was associated with the reduction of size of all fiber types in this muscle. Together, these findings confirm that age-related loss of muscle mass at 28 mo is strongly associated with the reduction in fiber size irrespective of fiber type, although these data are derived from studies of a muscle predominantly composed of type II fibers.

### Age-related mitochondrial membrane potential (ΔΨm) reduction in intact mitochondria of single isolated muscle fibers is associated with increased mitochondrial H_2_O_2_ production and oxidative damage

**Figure 2:**
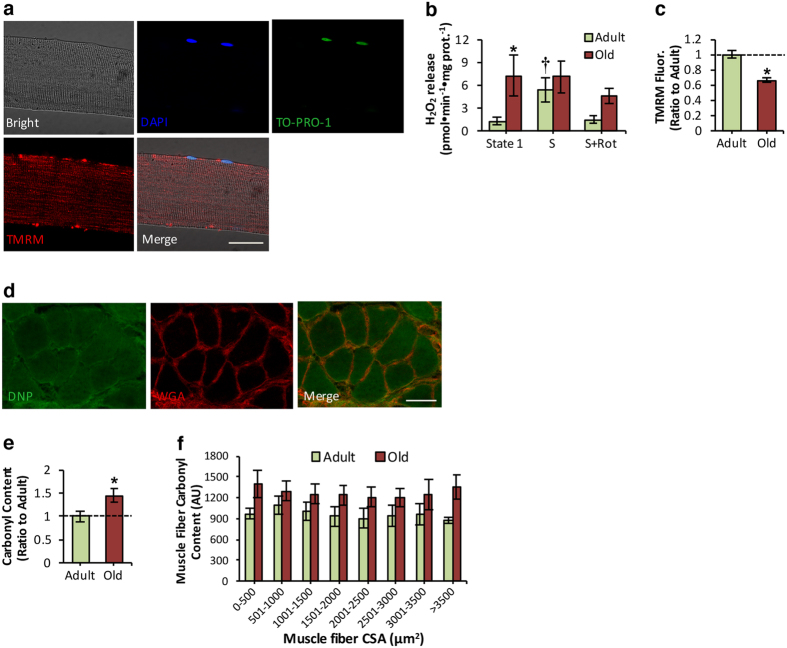
Age-related mtROS increase and oxidative damage reduce ΔΨm in intact mitochondria of single muscle fibers. (**a**) Confocal images of a saponin-permeabilized fiber isolated from the AT muscle under bright field, fluorescent image following loading with DAPI (1 μg/ml, blue), TO-PRO-1 iodide (200nM, Green), Tetramethylrhodamine, methyl ester (TMRM, 20nM, Red) and a merged image as indicated and analyzed by fluorescence microscopy. 60x original magnification. Scale bar, 30 μm. (**b**) Mitochondrial hydrogen peroxide (H_2_O_2_) production assessed in permeabilized fiber bundles prepared from AT muscle of adult and old mice. Mitochondrial substrates and inhibitors; Succinate (S, 10mM) and Rotenone (Rot, 1 μM) were added as indicated in figure. *P < 0.05 compared with values from adult mice, ^†^P < 0.05 compared with values from other conditions of adult mice, ANOVA with LSD *post hoc* test, n = 6–8 mice/group. (**c**) Measurement of mitochondrial membrane potential (ΔΨm) in intact mitochondria of isolated AT fibers from adult and old mice, assessed by TMRM fluorescence, Arbitrary Units (A.U). *P < 0.05 compared with values from the adult mice, Student’s *t*-test, unpaired, n = 14 fibers, 7 mice/group. (**d**) Transverse section of an AT muscle from an old mouse immune-labeled for dinitrophenyl (DNP, green), to evaluate protein carbonyl content, WGA (5 μg/ml, red), to visualize extracellular matrix, and a merged image as indicated and analyzed by confocal microscopy. Scale bar, 50 μm. (**e**) Protein carbonyl content in AT skeletal muscle of adult and old mice. *P < 0.05 compared with values from the adult mice, Student’s *t*-test, unpaired, n = 4 mice/group. (**f**) Carbonyl intensity distribution by fiber size of individual myofibers from AT muscle of adult and old mice, Arbitrary Units (A.U). n = 4 mice/group. Data represent mean ± s.e.m.

To assess whether the loss of muscle mass observed in old mice was associated with changes in mtROS, we determined H_2_O_2_ efflux from mitochondria in permeabilized myofibers from the AT muscle of adult and old mice. The use of permeabilized fibers *in situ* permitted assessment of all mitochondria within a muscle fiber and preserved mitochondrial structural interactions and morphology^28^,^37^. As illustrated in Fig. 2a, saponin-permeabilized fiber from the AT muscle stained positive for TO-PRO-1 iodide indicating that plasma membrane permeabilization did not appear to disturb the mitochondrial membrane potential (ΔΨm) as shown by tetramethylrhodamine, methyl ester (TMRM) fluorescence (Fig. 2a). Mitochondrial H_2_O_2_ emission was significantly higher in permeabilized fiber bundles from old mice compared with adult mice under state 1 respiration (Fig. 2b). Following addition of succinate, a complex II substrate, intact mitochondria from adult mice showed a significant increase in H_2_O_2_ release whereas bundles from old mice showed no detectable changes (Fig. 2b), implying an age-related lack of sensitivity of muscle fibers to respond to succinate. Succinate-induced H_2_O_2_ release in myobundles from adult mice was abolished by the complex I inhibitor, rotenone, suggesting a complex I-dependent superoxide increase by reverse electron transport^38^,^39^ in response to succinate. To examine if the age-related increase in mtROS altered ΔΨm, isolated AT muscle fibers from adult and old mice were loaded with TMRM cationic fluorophore and imaged. Data demonstrated a significant reduction in TMRM fluorescence with age, suggesting that skeletal muscle aging is associated with a reduction in ΔΨm (Fig. 2c). To determine whether age-related muscle atrophy was associated with oxidative damage, immunohistochemical analysis of protein carbonyl content was examined in AT muscle cryosections from adult and old mice (Fig. 2d). In agreement with previous reports from our laboratory^10^,^18^, total protein carbonyl content was significantly increased with aging (Fig. 2e). This accumulation of protein carbonyls in myofibers of old mice was irrespective of fiber size (Fig. 2f) indicating that fibres of all sizes were associated with increased protein oxidation. Thus, our data demonstrate that muscle aging is associated with elevated mtROS production, with concomitant increases in oxidative damage and altered mitochondrial membrane potential.

### Mito-targeted SS31 peptide attenuates the age-related increase in mtROS

**Figure 3:**
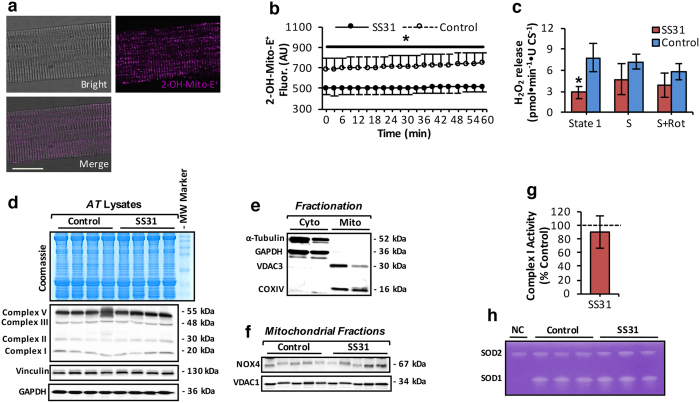
SS31 peptide attenuates mtROS increase that occurs with aging. (**a**) Representative images of a single fiber isolated from the AT muscle under bright field, fluorescent image following loading with MitoSOX Red (20nM, Purple), and a merged image as indicated and analyzed by confocal microscopy. 60x original magnification. Scale bar, 25μm. (**b**) Changes in mitochondrial 2-hydroxyethidium (2-OH-Mito-E^+^) fluorescence (normalized per CS activity) from isolated AT fibers of control old mice and old mice subjected to daily subcutaneous (SC) injections of mito-targeted SS31 peptide (SS31-treated mice) for 15 weeks (from 24 to 28 mo of age), Arbitrary Units (A.U). *P < 0.05 compared with values from SS31-treated old mice, ANOVA with LSD *post hoc* test, n = 6 mice/group. (**c**) Mitochondrial H_2_O_2_ production (normalized per CS activity) assessed in permeabilized fiber bundles prepared from AT muscle of control and SS31-treated old mice. Mitochondrial substrates and inhibitors; Succinate (S, 10mM) and Rotenone (Rot, 1 μM) were added as indicated in figure. *P < 0.05 compared with values from control old mice, Student’s *t*-test, unpaired, n = 6 mice/group. (**d**) Protein levels of oxidative phosphorylation (OXPHOS) complexes (I, II, III and V) from AT muscle of control and SS31-treated old mice. The intensity of the bands shown in the Coomassie brilliant blue-stained gel (top panel) was equivalent to glyceraldehyde 3-phosphate dehydrogenase (GAPDH) and Vinculin protein levels (bottom panel) and were used as loading controls. (**e**) Example western blot of α-Tubulin, GAPDH, voltage-dependent anion channel 3 (VDAC3) and cytochrome oxidase IV (COXIV) content to illustrate the purity of the extracted mitochondrial (Mito) and cytosolic (Cyto) skeletal muscle fractions. (**f**) Protein levels of NOX4 in skeletal muscle mitochondrial fractions of control and SS31-treated old mice. (**g**) Rotenone-sensitive respiratory chain complex I activity in AT skeletal muscle homogenates of control and SS31-treated old mice. n = 6 mice/group. (**h**) Native gels stained for SOD1 and SOD2 enzyme activities in AT skeletal muscle of control and SS31-treated old mice. Negative control (NC) included AT muscle lysate from Sod1 null mice. Data represent mean ± s.e.m.

To determine the effect of the mitochondria-targeted peptide on the age-related increase in mtROS, mice were subjected to daily subcutaneous (SC) injections of mito-targeted SS31 peptide for 15 weeks. SS-31 has previously been shown to reduce mitochondrial matrix superoxide in intact mitochondria of single isolated skeletal muscle fibers^33^ and C2C12 myotubes^34^. SS31 treatment attenuated the age-related change in mtROS as indicated by a significant reduction in MitoSOX Red fluorescence (Fig. 3a,b) and Amplex Red oxidation (Fig. 3c) in myofibers of 28 mo old mice to comparable levels to those of fibres of adult mice. We next examined the mechanism of SS31 peptide protection (Fig. 3d,f–h). Sources of mtROS identified in skeletal muscle include complex I, II, III^40^ and nicotinamide adenine dinucleotide phosphate oxidase 4 (NOX4)^19^,^33^. To assess mitochondrial levels of NOX4, mitochondrial and cytosolic fractions from skeletal muscle of control and SS31-treated mice were prepared (Fig. 3e). Reduced mtROS following treatment with SS31 was not related to changes in protein expression of complex I, II and III (Fig. 3d) or NOX4 (Fig. 3f). Similarly, no changes were observed in the enzymatic activities of respiratory complex I (Fig. 3g) or mitochondrial matrix superoxide dismutase 1 or 2 as assessed by native gels (Fig. 3h).

### MtROS determines the extent of oxidative damage and expression of redox-regulatory proteins in aging muscle

**Figure 4:**
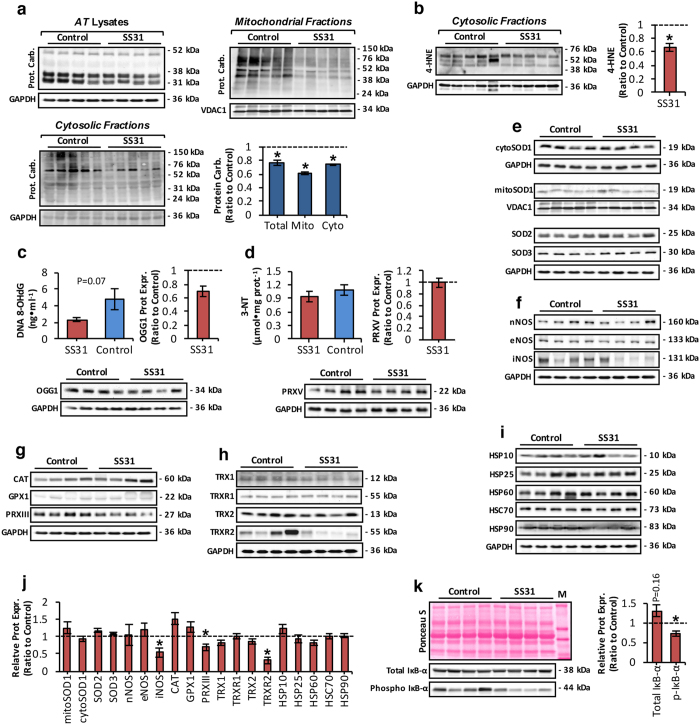
Age-related MtROS increase induces oxidative damage and alters the expression of redox-regulatory proteins. (**a**) Western blot analysis and quantification (bottom right panel) of protein carbonyls in mitochondrial (top right panel) and cytosolic (bottom left panel) skeletal muscle fractions, and AT lysates (top left panel) of control and SS31-treated old mice. *P < 0.05 compared with values from control mice. (**b**) Western blot analysis (left panel) and quantification (right panel) of 4-hydroxynonenal protein adducts (4-HNE) in cytosolic skeletal muscle fractions of control and SS31-treated old mice. *P < 0.05 compared with values from control mice. (**c**) Levels of 8-hydroxydeoxyguanosine (8-OHdG) in genomic DNA extracted from skeletal muscle (top left panel), and oxoguanine DNA glycosylase (OGG1) protein levels (bottom panel) of skeletal muscle from control and SS31-treated old mice and densitometric quantification of the blot (top right panel). n = 6 mice/group. (**d**) 3-nitrotyrosine (3-NT) content (top left panel), and peroxiredoxin V (PRXV) protein levels (bottom panel) of skeletal muscle from control and SS31-treated old mice and densitometric quantification of the blot (top right panel). n = 6 mice/group. (**e**) Representative western blots depicting superoxide dismutase (SOD) isoform expression in AT lysates and mitochondrial skeletal muscle fractions of control and SS31-treated old mice. (**f**) Protein expression levels of nitric oxide synthase (NOS) isoforms in AT lysates of control and SS31-treated old mice. (**g**) Western blots of the main H_2_O_2_ reducing enzymes including; catalase (CAT), glutathione peroxidase 1 (GPX1), and peroxiredoxin III (PRXIII) in AT lysates of control and SS31-treated old mice. (**h**) Protein expression of the main redox proteins involved in the thioredoxin-peroxiredoxin system including; thioredoxin 1 and 2 (TRX1 and TRX2), thioredoxin reductase 1 and 2 (TRXR1 and TRXR2) in AT lysates of control and SS31-treated old mice. (**i**) Western blots of heat shock proteins (HSPs) in AT lysates of control and SS31-treated old mice. (**j**) Densitometric analysis of the represented western blots shown in (**e**–**i**). *P < 0.05 compared with values from old control mice. (**k**) Effect of SS31- treatment on total and phosphorylated IκB-α (Phospho IκB-α) content (bottom left panel), and densitometric quantification of the blots (right panel). Ponceau S staining (top left panel) served as a loading control, molecular weight marker (M). *P < 0.05 compared with values from old control mice. Statistical differences between groups were determined using Student’s *t*-test, unpaired. Data represent mean ± s.e.m.

To determine if the significant reduction in mtROS, following treatment of old mice with SS31 results in reduced levels of age-related oxidative damage, changes in protein oxidation, lipid peroxidation, DNA damage and the expression of RONS regulatory proteins were examined (Fig. 4a–j). The reduction in mtROS - in old mice following treatment with SS31was associated with significantly reduced protein carbonyl content of skeletal muscle (Fig. 4a), a finding, which was slightly more pronounced in mitochondrial fractions (Fig. 4a). A significant reduction in protein carbonyl content was also evident in cytosolic fractions (Fig. 4a). To assess changes in lipid peroxidation, cytosolic fractions from skeletal muscle of control and SS31-treated old mice were immunoblotted for 4-hydroxynonenal (4-HNE) protein adducts (Fig. 4b). SS31-treatment reduced lipid peroxidation in muscles of old mice as indicated by a decline in 4-HNE content (Fig. 4b). Muscle from SS31-treated old mice showed no sigificant difference in 8-OHdG (Fig. 4c) but did demonstrate a reduction in protein expression of oxoguanine DNA glycosylase (OGG1), (Fig. 4c), a primary enzyme responsible for the excision of 7, 8-dihydro-8-oxoguanine lesions^41^. No changes were observed between control and SS31-treated old mice in the level of protein nitration (3-NT), (Fig. 4d) and the expression of a peroxynitrite reductase, peroxiredoxin V (PRXV), (Fig. 4d). Overall, these results suggest that attenuation of age-related mtROS increase was associated with a reduction in several indices of age-related oxidative damage. To determine whether reduced mtROS in muscle of the SS31-treated old mice was associated with adaptations in the levels of proteins involved in antioxidant defences, the levels of RONS regulatory proteins including; superoxide dismutase (SOD) isoforms (Fig. 4e), nitric oxide synthase (NOS) isoenzymes (Fig. 4f), H_2_O_2_ scavenging enzymes including glutathione peroxidase 1, catalase and PRXIII (Fig. 4g), redox proteins involved in the thioredoxin-peroxiredoxin system (Fig. 4h) and heat shock proteins (Fig. 4i) which have all been shown to provide protection against increased RONS production^7^,^10^ were determined. Densitometric quantification of the blots presented in Fig. 4e–i revealed a reduction in protein levels of PRXIII and thioredoxin reductase 2 (TRXR2 (Fig. 4j). Decreased protein levels of the inducible isoform of NOS (iNOS) were also observed in muscle tissue of the SS31-treated old mice compared to control old mice (Fig. 4j). The NFκB signalling pathway has been shown to regulate the expression of iNOS^42^ and antioxidant defence enzymes^5^ and we next tested whether the attenuation of age-related mtROS increase was associated with altered activation of the NFκB pathway which we have previously described as chronically activated in old mice^43^. Muscles of SS31-treated old mice demonstrated a significant reduction in phosphorylated IκB-α (Phospho IκB-α) suggesting a reduced activation of NFκB, although no difference in total IκB-α content was seen (Fig. 4k).

### Age-related mitochondrial redox changes are associated with compromised mitochondrial biogenesis and impaired mitophagy

**Figure 5:**
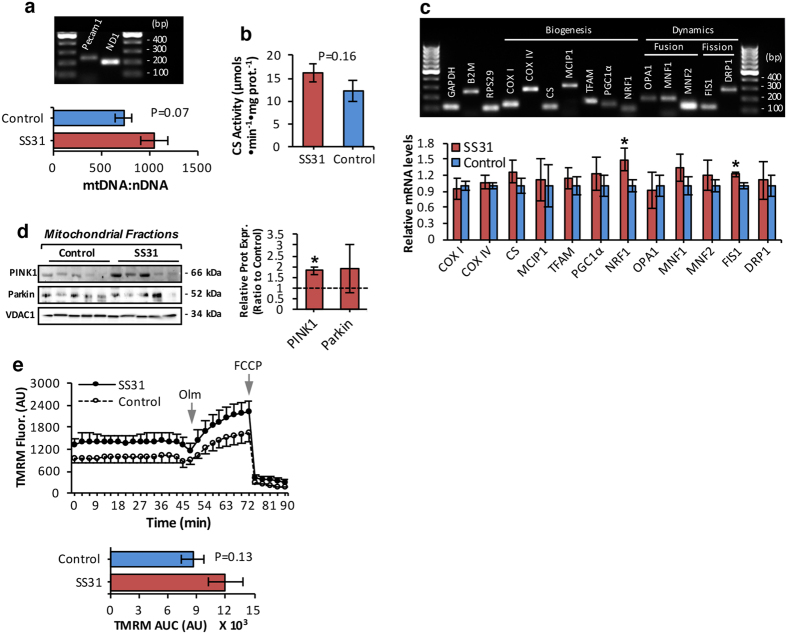
Reduced mitochondrial content and defective mitophagy are linked to age-related changes in mitochondrial redox homeostasis. (**a**) Agarose gel electrophoresis of the reverse transcriptase PCR (RT-PCR) amplification products of mitochondrial ND1 and nuclear Pecam 1 transcripts (top panel). Lanes 1 and 4, 100bp DNA molecular weight marker. Quantitative RT-PCR measurement of mitochondrial DNA (mtDNA), normalized to the amount of nuclear DNA (nDNA) in skeletal muscle of control and SS31-treated old mice (bottom panel). n = 6 mice/group. (**b**) Citrate synthase (CS) activity in skeletal muscle of control and SS31-treated old mice. n = 6 mice/group. (**c**) Representative image of agarose gel electrophoresis of the RT-PCR amplification products of GAPDH, B2M, RPS29, COXI, COXIV, CS, MCIP1, TFAM, PGC1α, NRF1, OPA1, MNF1, MNF2, FIS1, and DRP1 transcripts (top panel). Lanes 1 and 17, 100bp DNA molecular weight marker. The PCR products correspond to the amplicon sizes shown in Supplementary Table 1. Relative mRNA levels of genes involved in mitochondrial biogenesis and dynamics, analyzed by quantitative RT-PCR (bottom panel). mRNA levels were normalized against the housekeeping genes; GAPDH, B2M and RPS29. *P < 0.05 compared with values from old control mice, n = 6 mice/group. (**d**) Western blots of isolated mitochondrial fractions from skeletal muscle of control and SS31-treated old mice immunodetected for PTEN-induced putative kinase 1 (PINK1), and ubiquitin ligase Parkin mitophagy markers (left panel), and densitometric quantification of the blots (right panel). *P < 0.05 compared with values from old control mice. Statistical differences between groups were determined using Student’s *t*-test, unpaired. (**e**) Measurement of ΔΨm in intact mitochondria of isolated AT fibers from control and SS31-treated old mice, assessed by changes in TMRM (30nM) fluorescence in response to oligomycin (Olm, 2.5 μM) and the protonophore carbonylcyanide-p-trifluoromethoxyphenyl hydrazone (FCCP, 4 μM), added at the indicated time points (top panel). Statistical analysis of the area under the TMRM fluorescence trace (AUC) for control and SS31-treated old mice (bottom panel). n = 12 fibers, 6 mice/group. Data represent mean ± s.e.m.

We determined whether the reduced redox environment in muscle of SS31-treated old mice (Figs 3b,c and 4a–c) modified the age-related decrease in mitochondrial abundance^24^,^25^ (Fig. 5a–c). There was a tendency for SS31-treated mice to have an increased (p = 0.07) mitochondrial DNA (mtDNA) copy number per nuclear genome, (Fig. 5a) and increased (p = 0.16) citrate synthase activity (Fig. 5b), potentially demonstrating a higher mitochondrial content in muscle of SS31-treated old mice, although these changes did not reach significance. To understand at which step mitochondrial biogenesis may be affected, we measured the expression of genes involved in mitochondrial dynamics and biogenesis (Fig. 5c). Quantitative RT-PCR analysis showed that mRNA levels for the majority of the genes involved in mitochondrial biogenesis, fusion and fission tended to be higher in muscle of SS31-treated old mice compared with controls (Fig. 5c). Moreover, transcriptional level of nuclear respiratory factor-1 (NRF1), a transcription factor essential for the integration of nuclear and mitochondrial encoded gene transcription as well as mitochondrial fission-1 (FIS1), a mediator of mitochondrial division^44^, was significantly up-regulated in SS31-treated compared with control old mice (Fig. 5c). These data support the hypothesis that changes in mitochondrial redox homeostasis during aging (Figs 2b and 4a) are associated with altered skeletal muscle mitochondrial content by suppression of the expression of genes involved in mitochondrial dynamics and biogenesis. To test the ability of SS31 treatment to impact on impaired age-related mitophagic potential associated with reduced expression of ubiquitin ligase Parkin^27^, isolated mitochondrial fractions from skeletal muscle were immunoblotted for PTEN-induced putative kinase 1 (PINK1) and Parkin mitophagy markers (Fig. 5d). We observed increased recruitment of PINK1 in isolated muscle mitochondria of SS31-treated compared with control old mice (Fig. 5d), suggesting an increased mitophagic response, although no significant change in Parkin was observed. Moreover, ΔΨm of intact mitochondria of isolated AT muscle fibers, examined by changes in TMRM fluorescence in response to oligomycin and the protonophore FCCP (Fig. 5e) showed a trend towards an increase in mitochondria of SS31-treated old mice compared with control mice (Fig. 5e) although this did not reach significance (p = 0.13).

### Loss of muscle mass and force with age is not governed by age-related changes in mitochondrial redox potential

**Figure 6:**
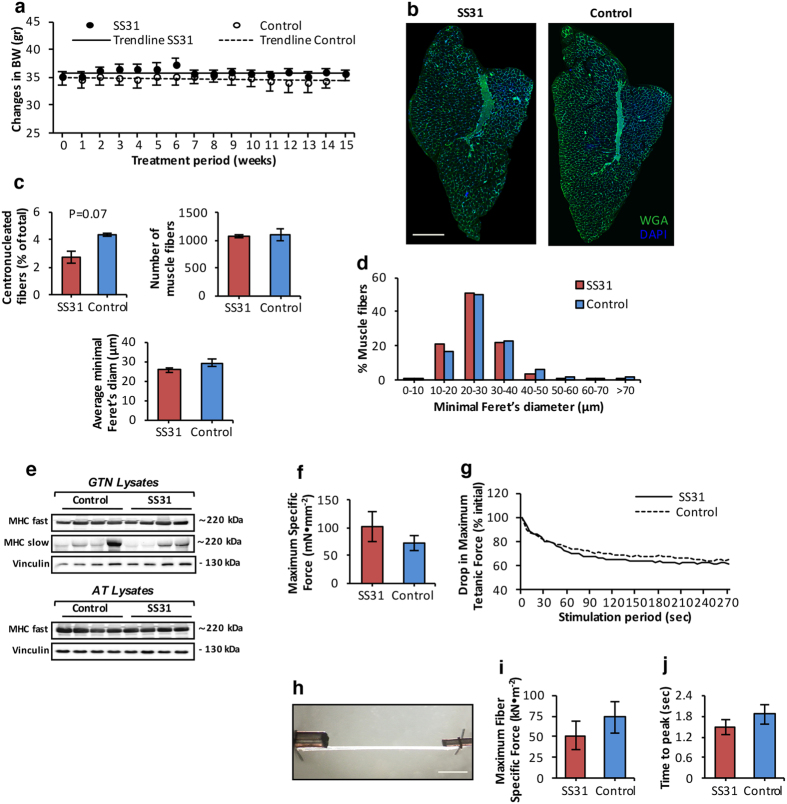
Mitochondrial redox homeostasis is not implicated in the processes of age-related muscle fiber atrophy. (**a**) Time course of the changes in mouse body weigh (BW) during the 15-week period of daily SC injections of mito-targeted SS31 peptide. Trend lines indicate no changes in BW during the treatment period, BW was monitored weekly. n = 6 mice/group. (**b**) Transverse sections of AT muscle from 28 mo old control and SS31-treated mice obtained following the 15-week treatment period, and stained with WGA (5 μg/ml, green), to visualize extracellular matrix, and DAPI (1 μg/ml, blue), to mark nuclei. Scale bar, 400μm. (**c**) Percentage of fibers showing centrally located nuclei in AT muscle of control and SS31-treated old mice (top left panel); total number of muscle fibers in AT muscle of control and SS31-treated old mice (top right panel); mean minimal Feret’s diameter of individual fibers from AT muscle of control and SS31-treated old mice (bottom panel). n = 6 mice/group. (**d**) Frequency distribution of fiber size of AT muscle from control and SS31-treated old mice. n = 6 mice/group. (**e**) Representative western blots of the fast (MHC fast) and slow (MHC slow) MHC content in gastrocnemius (GTN, top panel) and AT muscle (bottom panel) of control and SS31-treated old mice. (**f**) Maximum isometric specific force measured *in situ,* normalized to total fiber CSA of extensor digitorum longus (EDL) muscle from control and SS31-treated old mice. n = 6 mice/group. (**g**) *In situ* measurements of the drop in maximum isometric specific force of EDL muscle during a series of repeated isometric contractions (300ms, at 100Hz, every 5s), expressed as a percentage of the initial force. Lines represent the average response of 6 muscles, data are not significantly different between control and SS31-treated old mice. n = 6 mice/group. (**h**) Image of a skinned myofiber isolated from an AT muscle of a 28 mo old mouse, attached to a force transducer and high-speed length controller. Scale bar, 400 μm. (**i**) *Ex vivo* measurements of maximum fiber specific force normalized to fiber CSA of skinned myofibers isolated from the AT muscle of control and SS31-treated old mice. n = 30 fibers, 6 mice/group. (**j**) *Ex vivo* measurements of the time to peak maximum tension of skinned fibers isolated from the AT muscle of control and SS31-treated old mice. n = 30 fibers, 6 mice/group. Data represent mean ± s.e.m.

The data presented above indicate that treatment of old mice with mito-targeted SS31, resulted in modification, at least in part, of a number of indices of age-related mitochondrial oxidative damage, mitophagy and mitochondrial content. We determined whether the changes observed in mitochondrial redox environment in skeletal muscle of old mice treated with SS31were associated with changes in muscle fiber atrophy seen in muscles of 28 month old mice. Figure 6a demonstrated no change in Body Weight of mice during the 15-week SS31 treatment period compared with control old mice. Following treatment with SS31, muscle masses were not different for any of the muscles studied (Table 1), suggesting that SS31 did not alter the muscle fiber atrophy that occurred between 24 to 28 mo of age. To assess changes in muscle morphology, transverse sections of AT muscles from control and SS31-treated old mice were double immunolabeled with WGA, to visualize extracellular matrix, and DAPI, to mark nuclei (Fig. 1b). The number of centronucleated fibers was not changed in muscle of the SS31 treated compared with the control group (Fig. 6c). Similarly, the total number of fibers per AT muscle (Fig. 6c) and the average minimal Feret’s diameter (Fig. 6c) were not altered following the SS31 treatment. Further quantitative analysis of individual fiber size showed no differences in myofiber size (Fig. 6d) or fiber type distribution (Fig. 6e) between control and SS31-treated old mice. Thus no protective effect of SS31 treatment was seen on age-related muscle fiber atrophy and muscle mass. Functional measurements of extensor digitorum longus (EDL) muscle strength *in situ* revealed no differences in maximum isometric specific force between control and SS31-treated old mice (Fig. 6f). Similarly, *in situ* measurements of the decline in force of EDL muscles during a series of repeated isometric contractions were not altered in SS31-treated compared with control old mice (Fig. 6g). *Ex-vivo* force generation of single isolated skinned fibers (Fig. 6h) obtained from AT muscle was also assessed to explore the force generated by sarcomeric proteins independent of fiber number, innervation, ATP levels and calcium release^1^. Both, specific force (Fig. 6i) and time to peak maximum tension (Fig. 6j) did not differ between the control and SS31-treated old groups. Therefore, SS31 treatment had no effect on age-related loss of fiber or muscle mass and function.

## Discussion

The production of ROS by skeletal muscle mitochondria is important as it is proposed to underlie oxidative damage in several muscle pathologies^23^,^35^ and contributes to retrograde redox signalling from the organelle to the cytosol and nucleus^38^. Although cumulative oxidative damage has been proposed to induce age-associated decline in mitochondrial content and function^29^,^30^, this remains a controversial topic^31^,^32^. It is unclear whether age-associated mitochondrial redox changes are responsible for age-related muscle atrophy, associated with loss of muscle fibers and atrophy of muscle fibers which contribute to loss of muscle mass and function. In order to examine these fundamental questions we i) characterised the structural changes to muscle which may underlying sarcopenia and ii) assessed whether there was a functional link between mitochondrial redox homeostasis and age-related muscle atrophy by using a pharmacological intervention based on a novel peptide, targeted to the mitochondria. This approach included the use of mito-targeted SS31 peptide, based on recent reports from our laboratory^33^,^34^ and others^35^,^36^ which have shown that application of SS31 can attenuate mtROS in intact mitochondria of single isolated skeletal muscle fibers.

We showed that age-related alterations in the AT muscle of mice include a specific reduction in the numbers of fast glycolytic fibers (type IIb), which occurred at middle age (between the age of 10 to 19 month old mice), but did not immediately contribute to the sarcopenic phenotype due to the compensatory hypertrophy of the remaining muscle fibers. We further demonstrated that loss of muscle mass and strength observed in 28 month old compared with adult mice was attributed to a reduction in myofiber size irrespective of fiber type specificity, with little further reduction in fiber numbers. Our findings are in agreement with recent human studies, highlighting age-related atrophy of all fiber types as the underlying cause of sarcopenia^27^.

Many studies have utilized mechanically isolated mitochondria from skeletal muscle to study age-dependent changes in mtROS production^20^,^21^ and mitochondrial function^45^, a preparation that can either severely disturb mitochondrial structural integrity or lead to the loss of disrupted mitochondria during the isolation process, thus potentially over or underestimating mitochondrial function with aging^28^. Here we examined mitochondrial redox homeostasis and function *in situ,* utilizing permeabilized muscle fibers, isolated from skeletal muscle. This approach has significant advantages over mechanical isolation techniques, as it permits representation of all mitochondria within their native intracellular environment of the muscle fiber, thus preserving mitochondrial morphology and structural interactions with other sub-cellular compartments^28^,^37^. Consistent with our hypothesis, we provided evidence that muscle aging was associated with increased ROS production by intact mitochondria of muscle, shifting the cellular redox environment to a more oxidized state. We further show that attenuation of age-dependent mitochondrial redox changes in muscle by SS31 was associated with improvements in aspects of oxidative damage, mitophagy and mitochondrial content.

Previous studies in skeletal muscle have reported age-related decline in mitochondrial content^24^,^25^ and impaired mitophagy^27^. The role of redox signalling in regulating the autophagic system has recently been reported in mdx mouse model, a surrogate for Duchenne muscular dystrophy, in which genetic downregulation of NOX2 activity abrogated defective autophagy and lysosome biogenesis^46^. Here, we show for the first time that altered mitochondrial redox state is associated with defective mitophagy in skeletal muscle of old mice. Pharmacological attenuation of age-related mtROS increase resulted in improved mitophagy and partially prevented the decline in mtDNA abundance and biogenesis that occur in muscle aging. Thus, our present findings highlight the role of muscle mitochondrial redox potential in at least partially regulating gene expression, mitochondrial protein synthesis, enzymatic activity and ultimately mitochondrial integrity during muscle aging.

It is important to note that the current study examined muscle mitochondria, but it is possible that systemic treatment of mice with SS31 may have had effects on mitochondria from non-muscle tissue. It is particularly important to consider the potential effect on mitochondria of the motor neuron, since it has been proposed that redox homeostasis in motor neurons plays a key role in initiating sarcopenia during aging^7^.

The most striking conclusion in the current study is that age-related increase in muscle mtROS and the concomitant changes in oxidative damage and mitochondrial function were not associated with the later stages of muscle atrophy during the ageing process. Administration of SS31 failed to rescue age-related myofiber atrophy or improve muscle force generation in old mice. In support of this finding, previous reports from our laboratory^47^ and others^48^ have shown that heterozygous knockout of MnSOD (Sod2^+/−^) had no major effect on skeletal muscle structure or function. Both morphological appearance and contractile characteristics assessed via changes in maximum muscle tetanic force and following a period of repeated isometric contractions did not differ between WT and Sod2^+/−^ mice. Similarly, targeted overexpression of the human catalase gene to mitochondria (MCat mice) improved mitochondrial function but failed to rescue age-associated muscle fibrosis and loss of fiber size^2^. Hence, these data suggest that age-related muscle fiber atrophy is not attributable to age-dependent mitochondrial redox changes, associated with increased muscle mtROS and accumulated mitochondrial oxidative damage.

It is worth noting that we did not present evidence of localisation of the SS31 to the mitochondria, although evidence from other studies shows that SS31 rapidly and freely crosses the skeletal muscle plasma membrane and concentrates greater than 1000 fold in the mitochondria^49^. It is possible however that the presence of SS31 at much lower levels in other cellular regions may be contributing to any effects reported.

Another important finding is that use of a potential pharmacological agent targeted to the mitochondria at a relatively old age (24 mo old mice) can still modify ROS generation by skeletal muscle mitochondria. The majority of studies examining the contribution of muscle mtROS in age-related muscle mitochondrial function and function have relied on rodent models with genetic modifications of mitochondrial redox systems^2^,^23^,^47^. We therefore provide compelling evidence that the use of pharmacological targeted approaches can improve the change in mitochondrial redox homeostasis that occurs with aging with potential implications for the development of novel therapeutic strategies based on pharmacological targeting of other subcellular sources of ROS.

This is the first study to utilise a pharmacological approach, based on a mitochondria targeted antioxidant peptide and demonstrate that age-related changes in mitochondrial redox environment are associated with mitochondrial oxidative damage and mitophagic response, known to occur with advancing age. Moreover, whilst we demonstrate that muscle mitochondrial redox homeostasis plays a key role in the decline of mitochondrial organelle integrity that occurs with aging, it does not appear to be implicated in age-related muscle fiber atrophy. Our results have substantial translational implications for the use and/or development of muscle mitochondria-targeted treatment of human mitochondrial myopathies.

## Methods

### Chemicals and Reagents

Unless stated otherwise, all chemicals used in this study were obtained from Sigma Chemical Company.

### Mice

Male and female WT C57Bl/6 mice (8 mo old) were obtained from Charles River Laboratories (Margate, UK) and aged to 28 mo at the Biomedical Services Unit, University of Liverpool. All experiments were conducted on 9–10, 12, 17–18, 24 and 28 mo old mice in accordance with UK Home Office guidelines under the UK Animals (Scientific Procedures) Act 1986. Mice were fed a CRM (P) rodent diet and were maintained under barrier conditions in microisolator cages on a 12-h dark/light cycle. For simple tissue collection, mice were euthanized by cervical dislocation, and muscles/tissues were either rapidly removed, snap-frozen, and stored at −80 °C, or embedded in Tissue-Tek (VWR) and rapidly frozen in nitrogen-chilled isopentane for histological analysis. Mice subjected to *in situ* muscle force measurements were anesthetized with intraperitoneal (IP) injections of ketamine hydrochloride (66 mg/kg) and medatomidine hydrochloride (0.55 mg/kg), with supplemental injections provided to maintain an adequate level of anesthesia throughout the procedure. All procedures were approved by the University of Liverpool Animal Welfare and Ethical Review Body.

### SS31 administration

Mitochondria-targeted SS31 tetrapeptide was prepared by solid-phase peptide synthesis and purchased from Insight Biotechnology. SS31-treated mice received daily subcutaneous (SC) injections (neck and shoulder area) of SS31 (1.5 mg/kg; n = 8), starting at 24 mo of age and continuing until mice reached 28 mo of age (4-month treatment period). A separate group of animals was subjected to SC injections with saline (control group; n = 8). Mouse body weight was monitored on a weekly basis and SS31 dosage was adjusted accordingly. We selectively chose to administer mice with SS31 between the age of 24 to 28 mo old since preliminary studies (Fig. 1a) showed that the major loss of muscle mass occurred within that time period, primarily due to muscle fiber atrophy. The dosage of SS31 used in the present study was based on the dosage previously used in protection against immobilization-induced muscle atrophy^36^ and in a model of skeletal muscle insulin resistance^35^; no toxicity has previous been observed with doses as high as 50 mg/kg^50^.

### *In situ* muscle function analysis

Extensor digitorum longus (EDL) muscle contractile properties were measured *in situ* as previously described^6^,^51^. To assess the maximum isometric tetanic force (P_o_) of the EDL muscle, the distal tendon from anesthetized mice was severed and secured to the lever arm of a servomotor (Aurora Scientific). The knee of the hindlimb was fixed, the peroneal nerve was exposed, and bipolar platinum wire electrodes were placed across the nerve. Muscle optimal length (L_o_) was determined using a series of 1 Hz stimulation and set at the length that generates the maximal force. For determination of P_o_, EDL muscles were electrically stimulated to contract at L_o_ and optimal stimulation voltage (8–10 V) at 2 min intervals for 300 ms with 0.2 ms pulse width. P_o_ was assessed by increasing the frequency of stimulation from 10 to 50 Hz and, subsequently, in 50 Hz increments to a maximum of 300 Hz. P_o_ was identified when the maximum force reached a plateau, despite increasing stimulation frequency. After identification of P_o_, mice were subjected to a repetitive tetanic fatiguing protocol, which consisted of 60 consecutive isometric contractions (300 ms at 100 Hz every 5 sec for 5 min)^52^. The force deficit was calculated as the difference between a given P_o_ and the initial P_o_ for the same muscle, expressed as a percentage of the initial P_o_. Following completion of the procedures, mice were killed by cervical dislocation, and muscles/tissues were rapidly removed. Muscle fiber length (L_f_) and weight of EDL muscles were measured *ex vivo* to determine muscle cross-sectional area (CSA). Specific P_o_ (mN/mm^2^) was calculated by dividing P_o_ by total fiber CSA for each muscle.

### Muscle structure analysis

Anterior tibialis (AT) muscles were cryosectioned at −20 °C through the mid-belly with a thickness of 12 μm, and fluorescent immunohistochemical (IHC) staining was initiated the same day. Sections were rinsed with Phosphate Buffered Saline (PBS) and permeabilized in 0.2% Triton X-100 in PBS for 5 min. Fluorescein labelled wheat germ agglutinin (Vector Laboratories, 5 μg/ml) was used to identify extracellular matrix. Nuclei were identified using 4′,6-diamidino-2-phenylindole (DAPI, 1 μg/ml). Cross sections from 4–6 muscles/treatment group were examined by blinded observers to count the total number of fibers, percentage of centronucleated fibers and minimal Feret’s diameter. The minimal Feret’s diameter is the morphometric parameter that changes the least with the orientation of the sectioning angle and thus was used to avoid biased results. To ensure that all fibers/section were analysed, consecutive images acquired from each cryosection at 10x magnification were merged into a single high-resolution image using Adobe photoshop CS5 (see Fig. 1b). Image J software was used to analyze individual muscle fibers.

### Immunofluorescence analysis of MHC expression

Muscle cross sections (10 μm thick) were immunolabeled for myosin heavy chain (MHC) isoform expression; MHC I, MHC IIa, MHC IIx and MHC IIb using a previously described method^27^,^53^. Sections were rinsed with PBS and blocked using goat serum (12% in PBS) for 75 min at room temperature. Primary antibodies specific for skeletal muscle MHC were purchased from Sigma; anti-MHC I (M8421, 1:500), and the Developmental Studies Hybridoma Bank (DSHB, University of Iowa); anti-MHC IIα (SC-71, 1:200), anti-MHC IIx (6H1, 1:100), and anti-MHC IIβ (BF-F3, 1:150). Antibodies were diluted in 12% goat serum and incubated for 2 h at room temperature. Sections were washed with PBS 3 × 5 min, followed by incubation with appropriate secondary antibodies (Alexa Fluor 350, 488 and 532; Invitrogen; diluted 1:600) for 1 h at room temperature. Slides were mounted using ProLong Diamond antifade reagent (Life Technologies) and cured for 16 hours before imaging.

Fluorescence images were obtained using a C1 confocal laser scanning microscope (Nikon) equipped with a 405 nm excitation diode laser, a 488 nm excitation argon laser, and a 543 nm excitation helium-neon laser. Emission fluorescence was detected through a set of 450/35, 515/30, and 605/15 emission filters. Using 10x magnification, fluorescence images were captured and analyzed with the EZC1 V.3.9 (12 bit) acquisition software. Individual images acquired from each section were merged into a single high-resolution image using Adobe Photoshop CS5 (see Fig. 1e,f).

### Preparation of permeabilized muscle fiber bundles

Selective plasma membrane permeabilization of fiber bundles was performed according to methods described by Kuznetsov *et al*.^37^. Application of this technique to analyse intact skeletal muscle mitochondria *in situ* has been thoroughly described^28^,^35^,^54^. In brief, AT muscles were placed in ice-cold buffer A, containing (in mM) 50 K-MES, 7.23 K_2_EGTA, 2.77 CaK_2_EGTA, 20 imidazole, 0.5 DTT, 20 taurine, 5.3 Na_2_ATP, 15 PCr, and 6.56 MgCl_2_-6H_2_O (pH 7.3 at 4 °C) and trimmed of connective tissue and fat. Muscles were manually teased into small bundles of fibers and treated with 50 μg/ml saponin (in buffer A) for 30 min at low rocking speed. Saponin specifically interacts with the cholesterol-rich muscle fiber plasma membrane while keeping mitochondrial membranes (which have a low cholesterol content) completely intact^37^. Following permeabilization, fiber bundles prepared for mitochondrial H_2_O_2_ emission measurements, were washed 3 × 10 min in ice-cold buffer Z, containing (in mM) 110 K-MES, 35 KCl, 1 EGTA, 5.3 Na_2_ATP, 10 K_2_HPO_4_, and 3 MgCl_2_-6H_2_O (pH 7.3 at 4 °C), supplemented with 5 mg/ml BSA. Permeabilized fiber bundles prepared for respiration analyses were washed 3 × 10 min in ice-cold buffer B containing, (in mM) 100 K-MES, 7.23 K_2_EGTA, 2.77 CaK_2_EGTA, 20 imidazole, 0.5 DTT, 20 taurine, 3 K_2_HPO_4_, and 1.38 MgCl_2_-6H_2_O (pH 7.3 at 4 °C), supplemented with 2 mg/ml BSA.

### *Ex vivo* single muscle fiber analysis

Single muscle fibers isolated from the AT muscle were maintained in ice-cold relax solution, containing (in mM) 4.5 MgATP, 1 free Mg^2+^, 10 imidazole, 2 EGTA, and 100 KCL (pH 7.0 at 4 °C)^55^. Fibers were treated with 50 μg/ml saponin (in relax solution) for 15 min on ice and then mounted onto an 802D permeabilized fiber apparatus (Aurora Scientific). Skinned myofibers were attached to insect pins affixed to a 403A (5 mM) force transducer and 312C length controller, using ultra fine nylon thread (see Fig. 7h). Fibers were maintained in relax solution throughout assembly. Sarcomere length (SL) was measured using 900B Video Sarcomere Length (VSL) software (Aurora Scientific). SL was adjusted on each fiber to 2.4–2.6 μm, fiber diameter was measured at four intervals along the length, and a circular circumference assumed for the basis of CSA calculation. Fibers were maximally activated in Ca^2+^ activating solution (pCa 4.5), containing in addition to Ca^2+^. (in mM) 5.3 MgATP, 1 free Mg^2+^. 20 imidazole, 7 EGTA, 19.6 PCr, and 64 KCl (pH 7.0 at 4 °C). Peak force was recorded and normalised to fibre CSA^55^.

### Mitochondrial H_2_O_2_ emission measurements

Mitochondrial H_2_O_2_ production was measured using the Amplex Red-horseradish peroxidase (HRP, Molecular Probes) method as previously described^20^,^56^. Fluorescence was monitored using a microplate fluorometer (FLUOstar Optima, BMG) at an excitation/emission wavelength of 544/590 nm. Permeabilized fiber bundles were incubated in buffer Z containing Amplex Red (80 μM) and HRP (1U/ml) at 37 °C. CuZnSOD (37.5U/ml) was also added to convert any superoxide present into H_2_O_2_. The slope of the increase in fluorescence was converted to the rate of H_2_O_2_ production with a standard curve, generated in the absence of samples using successive addition of known H_2_O_2_. The rates of H_2_O_2_ production were determined in the absence of substrates (State 1) or in the presence of mitochondrial substrates and inhibitors; Succinate (S, 10 mM) and Rotenone (Rot, 1 μM). At the conclusion of each experiment, bundles were placed in liquid N_2_ and stored at −80 °C for assessment of total protein content. H_2_O_2_ production is expressed as picomoles per minute per milligram protein and as per unit of Citrate Synthase (CS) activity.

### Mitochondrial DNA quantification

Mitochondrial DNA (mtDNA) was measured by quantitative RT-PCR (qRT-PCR) as described by Chen *et al*.^57^. Genomic DNA (gDNA) was extracted, RNase-treated and purified (Purelink Genomic DNA kit, Invitrogen) from skeletal muscle, and diluted to 2.5 ng/μl. Quantification of mtDNA present per nuclear genome was determined by using the following primer pairs: mitochondrial ND1 forward primer, CCTATCACCCTTGCCATCAT; mitochondrial ND1 reverse primer GAGGCTGTTGCTTGTGTGAC and Pecam1 gene on chromosome 6 nuclear DNA forward primer, ATGGAAAGCCTGCCATCATG; nuclear Pecam1 gene on chromosome 6 reverse nuclear DNA primer, TCCTTGTTGTTCAGCATCAC. Quantification of relative copy number differences was carried out by analyzing the difference in threshold amplification between mtDNA and nuclear DNA (delta delta C(t) method).

### DNA damage

DNA damage was assessed by the content of 8-hydroxydeoxyguanosine (8-OHdG) as described recently by Changou *et al*.^58^. Extracted gDNA (1 μg) was converted to single-stranded DNA by incubating the sample at 95 °C for 5 min and rapidly chilling on ice. DNA samples were digested to nucleosides by incubating the denatured DNA with nuclease P1 (5U) for 2 h at 37 °C in sodium acetate (20 mM) pH 5.2, followed with treatment of alkaline phosphatase (5U, New England Biolabs) for 1 h at 37 °C in Tris (100 mM), pH 7.5. The reaction mixture was centrifuged for 5 min at 6,000 g and the supernatant was used for the 8-OHdG DNA damage ELISA kit (Cell Biolabs) according to supplier’s protocol.

### Fluorescence-based methods to measure MitoSOX Red oxidation and mitochondrial membrane potential

To monitor changes in mitochondrial superoxide, isolated fibers from the AT muscle were loaded with MitoSOX Red (250 nM, Invitrogen) for 30 min as previously described^33^,^59^. Fibers were maintained in buffer Z containing MitoSOX Red (20 nM) during the experimental period. MitoSOX Red is a derivative of dihydroethidium designed for highly selective detection of superoxide in mitochondria and exhibits fluorescence (Fig. 3a) on oxidation and subsequent binding to mitochondrial DNA^60^. The reaction between superoxide and MitoSOX Red generates a highly specific red fluorescent product, 2-hydroxyethidium (2-OH-Mito-E^+^)^61^, monitored at an excitation/emission wavelength of 405/605 nm (see Fig. 3a). Measurement of mitochondrial membrane potential (ΔΨm) in intact mitochondria of isolated AT fibers was assessed by tetramethylrhodamine, methyl ester (TMRM, 30 nM, Invitrogen) fluorescence at an excitation/emission wavelength of 543/605 nm (see Fig. 2a). Changes in ΔΨm were determined in the presence of oxidative phosphorylation inhibitors; oligomycin (Olm, 2.5 μM) and FCCP (4 μM). Application of the technique to the study of TMRM changes in isolated skeletal muscle fibers has been reported by Irwin *et al*.^62^.

### Enzymatic activity assays

Enzymatic activity of CuZnSOD and MnSOD was assessed in native gels, with negative staining, as described previously^7^,^9^. Respiratory chain complex I activity in skeletal muscle homogenates was examined by the reduction of 2,6-dichloroindophenol (DCIP), followed spectrophotometrically at 600 nm as described by Janssen *et al*.^63^.

### Quantitative RT-PCR analysis

RNA from skeletal muscle was extracted, DNase-treated and purified using Direct-zol RNA miniprep (Zymo Research). Purified RNA was utilised to generate first-strand cDNA using the iScript cDNA Synthesis kit (Bio-Rad). Primers for qRT-PCR analyses are shown in Supplementary Table 1 and the optimal annealing temperature for each primer set was determined by using an annealing temperature gradient between 55 and 62 °C. Real-time PCR reactions were performed on an iCycler Detection System (Bio-Rad) using iQ SYBR Green Supermix (Bio-Rad). Specificity of the PCR products was determined by melt curve analysis and agarose gel electrophoresis. Three reference genes including GAPDH, beta-2-microglobulin (B2M) and ribosomal protein S29 (RPS29) were used as internal controls.

### Immunoblotting

Protein extracts (20 μg/sample) were separated using a standard protocol for western blots^8^. Peroxidase activity was detected using an ECL kit (Amersham International), and band intensities were analysed using Quantity One Software (Bio-Rad). Detailed information about antibodies and dilution can be found in Supplementary Table 2. Mitochondrial and cytosolic sub-cellular fractions were obtained from skeletal muscle as previously described^64^,^65^.

### Measurement of protein carbonyls

Protein carbonyls were analyzed by immunohistochemical and immunoblotting techniques. Protein carbonyl content in muscle lysates containing 20 μg protein was assessed with the Oxiselect protein carbonyl Immunoblot kit (Cell Biolabs). Intensities of 2,4-dinitrophenyl (DNP)-modified proteins were quantified by densitometry. Cryosections (10 μm thick) from AT skeletal muscle were immunolabeled for DNP following derivatization as described by Thomas *et al*.^66^. Protein carbonyl intensity was measured by confocal laser scanning microscopy.

### Statistical Analyses

Data are presented as mean ± SEM for each experiment. Statistical tests were used as described in the figure legends. Statistical analyses for potential differences between groups were determined using analysis of variance (ANOVA) followed by the *post hoc* LSD test. Single comparisons between two experimental conditions were undertaken using the unpaired Student’s t test. Data were analysed using SPSS 22 and p values of less than 0.05 were considered statistically significant.

## Additional Information

**How to cite this article**: Sakellariou, G. K. *et al*. Mitochondrial ROS regulate oxidative damage and mitophagy but not age-related muscle fiber atrophy. *Sci. Rep.* **6**, 33944; doi: 10.1038/srep33944 (2016).

## Figures and Tables

**Table 1:** Comparison of tissue weights from control and SS31-treated mice.

**Tissue**	**SS31**	**Control**
BW (gr)	35.4 ± 0.8	33.9 ± 1.3
AT (mg)	42.5 ± 3.2	40.7 ± 2.6
EDL (mg)	9.5 ± 0.7	9.6 ± 0.6
GTN (mg)	140.8 ± 9.9	145.7 ± 10.8
SOL (mg)	8.8 ± 0.3	9.3 ± 0.3
Liver (gr)	1.75 ± 0.06	1.83 ± 0.13
Spleen (mg)	203.2 ± 38.8	237.5 ± 78.8
Kidney (mg)	249.3 ± 12.4	242 ± 9.9
Heart (mg)	189.5 ± 13	197.7 ± 5.8
Lung (mg)	196.5 ± 20.3	197.5 ± 6.6
Brain (mg)	469.5 ± 4.2	484.7 ± 15

Values are presented as the mean ± SEM; n = 6 mice/group.
